# Successful induction of deep hypothermia by isoflurane anesthesia and cooling in a non-hibernator, the rat

**DOI:** 10.1186/s12576-021-00794-1

**Published:** 2021-03-30

**Authors:** Hiroki Shimaoka, Takahiko Shiina, Hayato Suzuki, Yuuki Horii, Kazuhiro Horii, Yasutake Shimizu

**Affiliations:** 1 Department of Basic Veterinary Science, Laboratory of Physiology, The United Graduate School of Veterinary Sciences, Gifu University, 1-1 Yanagido, Gifu, 501-1193 Japan; 2 Laboratory of Veterinary Physiology, Faculty of Applied Biological Sciences, 1-1 Yanagido, Gifu, 501-1193 Japan; 3 Center for Highly Advanced Integration of Nano and Life Sciences (G-CHAIN), Gifu University, 1-1 Yanagido, Gifu, 501-1193 Japan

**Keywords:** Anesthesia, Hibernation, Hypothermia, Organ injury, Thermoregulatory center

## Abstract

The aim of the present study was to establish a novel method for inducing deep hypothermia in rats. Cooling rats anesthetized with isoflurane caused a time-dependent decrease in rectal temperature, but cardiac arrest occurred before their body temperature reached 20 °C when isoflurane inhalation was continued during the cooling process. Stopping inhalation of isoflurane when the rectal temperature reached 22.5 °C successfully induced deep hypothermia, although stopping the inhalation at 27.5 °C resulted in spontaneous recovery of rectal temperature. The hypothermic condition was able to be maintained for up to 6 h. A large number of c-Fos-positive cells were detected in the hypothalamus during hypothermia. Both the maintenance of and recovery from hypothermia caused organ injury, but the damage was transient and recovered within 1 week. These findings indicate that the established procedure is appropriate for inducing deep hypothermia without accompanying serious organ injury in rats.

## Background

Therapeutic hypothermia has been suggested to be the most potent neuroprotective treatment for anoxic brain injury, cardiac arrest, and neonatal hypoxic–ischemic encephalopathy [1–4]. It is generally accepted that the therapeutic effects of hypothermia are mainly due to suppression of metabolic rate [2, 4]. Since cerebral metabolic rate decreases by 5% for each 1 °C reduction in body temperature [5], it is reasonable to expect that neuroprotection is stronger at lower temperatures. However, the common target temperature of therapeutic hypothermia is 32–34 °C [1–4]. The basis for this apparent inconsistency is considered to be related to a high incidence of adverse events by reducing body temperature to levels below 30 °C [6, 7]. For example, cardiac arrhythmias often occur below temperatures of 30 °C [7, 8]. Thus, the potential benefit of deep hypothermia is not adaptable to clinical application unless the adverse effects of deep hypothermia are substantially eliminated.

Expression of the beneficial properties without accompanying adverse events in a deep hypothermic condition would be feasible. Such hypothermia is typically observed during hibernation. Hibernation is characterized by repeated long periods of torpor, which is frequently interrupted by short periods of arousal [9–11]. During torpor, body temperature drops to only a few degrees above ambient temperature [9–11]. Nevertheless, obvious signs of organ injury are scarcely observed [12–15]. The ability of hibernators to maintain deep hypothermia in the absence of adverse events has the potential for translation into novel therapies for ischemia diseases [12–15]. Therefore, induction of deep hypothermia in homothermal animals that do not normally undergo hibernation is of particular interest [12–15].

Several lines of evidence indicate that adenosine A1 receptors (A1AR) within the central nervous system play an essential role in the entrance into torpor [16]. In accordance with this, it has been shown that intracerebroventricular (icv) injection of an A1AR agonist induces hibernation-like hypothermia not only in hibernators including ground squirrels and hamsters [17, 18] but also in a non-hibernator, the rat [19, 20]. Accordingly, the central A1AR can be considered as a target for induction of therapeutic hypothermia. However, the procedure for icv injection is likely to be a methodological limitation for clinical application.

The aim of the present study was to establish a novel method for inducing deep hypothermia, resembling hypothermia during hibernation, in rats. Anesthesia is useful for reducing body temperature, but deep hypothermia under an anesthetic condition causes cardiac arrest. Hence, we used inhalation anesthesia to induce hypothermia by taking advantage of the quick offset of its effects. Our results showed that removal of anesthetics at the appropriate timing is able to induce hypothermia without accompanying serious organ injury.

## Methods

### Animals

Male Sprague–Dawley rats (*Rattus norvegicus*, 8–12 weeks of age, 250–400 g in weight) were obtained from Japan SLC (Shizuoka, Japan). The rats were supplied with both laboratory chow (MF: Oriental Yeast, Tokyo, Japan) and water ad libitum*.* They were kept in plastic cages at 22 °C with a 12:12-h light and dark cycle prior to experiments. The experimental procedures were performed according to the guidelines for the care and use of laboratory animals approved by the Animal Care and Use Committee of Gifu University (permission numbers: 15096, 17014, 17167, H30-181, 2019-244, 2020-120).

### Induction of hypothermia by isoflurane inhalation and cooling

The rats were anesthetized by isoflurane inhalation (concentration: 2%). Isoflurane inhalation solution (Pfizer, NY, USA) was vaporized by a vaporizer (Natsume Seisakusho, Tokyo, Japan). To induce hypothermia, the anesthetized rats were placed in a refrigerator kept at an ambient temperature of 4 °C immediately after setting the recording devices for an electrocardiogram (ECG) and body temperature. In some experiments, inhalation of isoflurane was diminished and stopped when body temperature had dropped to the indicated temperature (see “Results”).

To examine the effects of maintaining deep hypothermia, the rats were placed on a cold stainless plate in the prone position when the rectal temperature had reached 15 °C. The temperature of the stainless plate was controlled at around 14 °C using a thermostat bath (CTP-101: TOKYO RIKAKIKAI, Tokyo, Japan) to maintain the rectal temperature at 15 °C. The hypothermic condition was maintained for the indicated time (see “Results”). If it was necessary to recover body temperature, the hypothermic rats were warmed by changing the temperature of the stainless plate to 37 °C.

Rectal temperature was measured by inserting a small thermistor (RET-2: Physitemp Instruments, NJ, USA) 5 cm into the rectum from the anus.

To investigate the involvement of the autonomic nervous system in induction and maintenance of deep hypothermia, we injected hexamethonium chloride (WAKO) intravenously (5 mg/kg) to block sympathetic/parasympathetic ganglionic transmission. For intravenous injection of hexamethonium chloride, the femoral vein was cannulated.

### ECG recording

An ECG was recorded from cable lead electrodes (OA213-067: Unique Medical, Tokyo, Japan) placed at the right forelimb and left hindlimb with a ground electrode placed at the right hindlimb. The signal was amplified by an amplifier (DAM50: World Precision Instruments, FL, USA) and recorded by a PowerLab system (AD Instruments, Bella Vista, NSW, Australia). Heart rate, PR interval, QRS duration and QT interval were calculated from raw ECG data with Lab Chart Pro (AD Instruments).

### Biochemical analyses

Blood was collected from the subclavian vein, and aspartate aminotransferase (AST), alanine aminotransferase (ALT), blood urea nitrogen (BUN), and lactate dehydrogenase (LDH) were measured by a veterinary automatic dry-chemistry analyzer (DRI-CHEM 3500 V: FUJIFILM, Japan). Blood collection was performed chronologically as follows: before induction of hypothermia (control), when the rectal temperature had reached 15 °C, after maintaining hypothermia for 3 h or 6 h, and immediately after recovery of body temperature and also 1 week later. The blood samples were immediately centrifuged (4 °C, 1200 × g, 10 min), and the obtained plasma was preserved at −30 °C before measurements.

### Immunohistochemistry

**Fig. 1:**
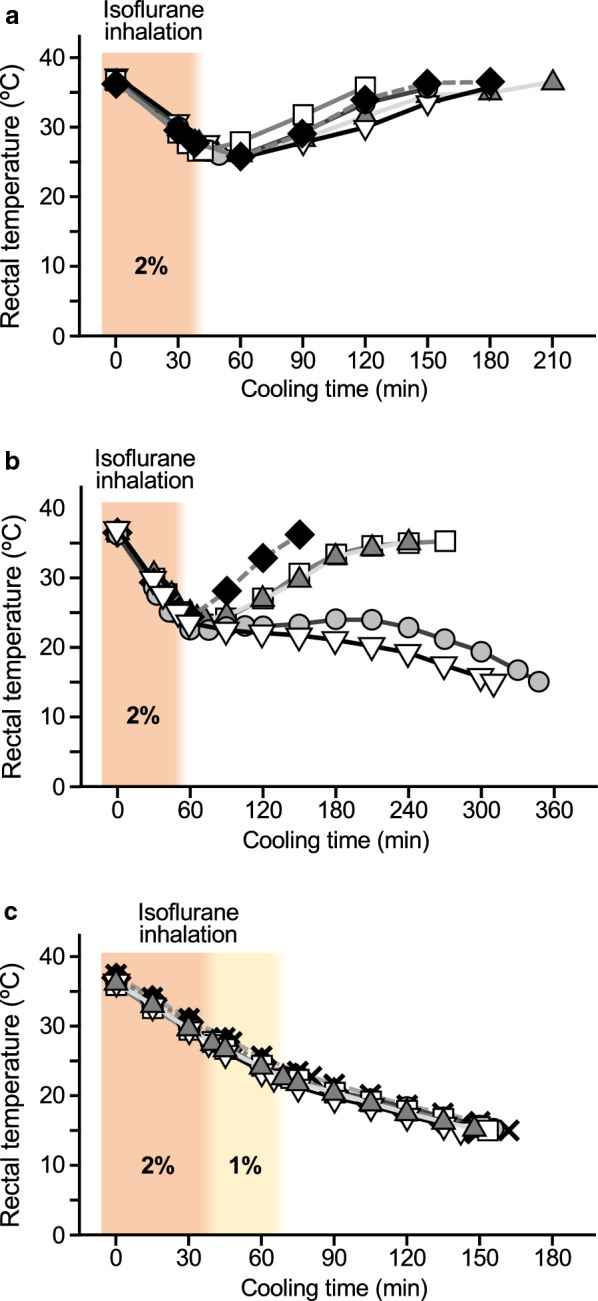
Temporal change in rectal temperature during induction of hypothermia by isoflurane inhalation and cooling in rats. Rats anesthetized with isoflurane were cooled by placing them in a cold room kept at 4 °C, and changes in rectal temperature were measured. **a** When the isoflurane inhalation was stopped at 27.5 °C, rats showed shivering spontaneously and rectal temperature increased. **b** When isoflurane inhalation was stopped at 25 °C, rectal temperature recovered spontaneously in three of five rats, whereas rectal temperature of the other two rats continuously decreased and eventually reached 15 °C. **c** Isoflurane concentration was kept at 2% until rectal temperature reached 27.5 °C and was then reduced to 1%, and then isoflurane inhalation was stopped at 22.5 °C. Using this procedure, the rectal temperature continuously decreased and reached 15 °C. Changes in rectal temperature in each rat (*n* = 5 for panels A and B, *n* = 6 for panel C) are shown

Artificial hypothermia was induced in rats by a procedure established in the present study (see Fig. 1c). When body temperature reached about 15 °C, the rats were anesthetized again with isoflurane and transcardially perfused with Ringer’s solution (200 mL) followed by 4% paraformaldehyde in 0.1 M phosphate buffer (pH 7.4; 200 mL). Rats under the active condition and euthermic rats anesthetized with isoflurane inhalation (2%) for 2 h were used as active control rats and anesthetized control rats, respectively. Tissues were isolated and post-fixed at 4 °C overnight. For cryostat sections, the specimens were rinsed in phosphate-buffered saline (PBS; pH 7.4) and stored in 30% sucrose in PBS at 4 °C overnight. They were then frozen with OCT compound medium (Sakura Finetech Japan, Tokyo, Japan). Cryostat sections were transversely cut at 50 µm in thickness. Consecutive sections were rinsed with 0.1 M PBS (3 × 10 min each). Free-floating sections were then put in a blocking solution of 0.5% Triton X-100 in PBS containing non-immune donkey serum (1:200) to prevent non-specific binding. After incubation in the blocking solution for 30 min, the sections were rinsed in PBS (3 × 10 min each) and incubated with a primary antibody directed against c-Fos (1:1000 dilution, Cat#ABE457, Sigma-Aldrich, MO, USA) at 4 °C overnight. After washing in PBS (3 × 10 min each), the sections were incubated for 2 h at room temperature with Cy3-conjugated donkey anti-rabbit IgG (1:400, Jackson ImmunoResearch, PA, USA) and then washed in PBS (3 × 10 min each). The sections were then mounted on gelatin-coated slides and coverslipped with Fluoromount (Diagnostic BioSystems, CA, USA).

### Cell counting

Acquired digital images were analyzed for c-Fos-positive cell counting. We examined five sections at intervals of 50 µm (every second section of 50-µm-thick sections) throughout the ventromedial preoptic nucleus (VMPO) (0.2 mm to −0.3 mm anterior–posterior) according to the rat brain atlas [21] in each animal. Fluorescence-positive cells were counted unilaterally. We calculated mean values for fluorescence-positive cells in the five sections in each animal.

### Statistics

All values are presented as means ± standard deviation (SD). Statistical difference between the two groups was determined by the paired *t* test. The values of blood biochemistry parameters were analyzed by one-way analysis of variance (ANOVA) or two-way ANOVA followed by Tukey’s post hoc test. Statistical significance was considered at *p* < 0.05.

## Results

### Induction of deep hypothermia by isoflurane inhalation and cooling in rats

Rats that had been anesthetized with isoflurane were cooled by placing them in a cold room kept at 4 °C, and changes in rectal temperature were measured. When inhalation of isoflurane at a concentration of 2% was continued throughout the cooling process, rectal temperature declined in a time-dependent manner, but cardiac arrest occurred before body temperature reached 20 °C (data not shown). In contrast, when the isoflurane inhalation was stopped at 27.5 °C, the rats showed shivering spontaneously and rectal temperature increased even though they remained under the condition of a low ambient temperature (Fig. 1a). When isoflurane inhalation was stopped at 25 °C, rectal temperature spontaneously recovered in three of five rats (Fig. 1b). The rectal temperature of the other two rats continuously decreased and eventually reached 15 °C (Fig. 1b).

The fact that deep hypothermia could be induced in some rats by stopping isoflurane inhalation prompted us to investigate the appropriate procedure for constant induction of artificial hypothermia. As a result of trial and error, we were able to establish the procedure as follows: isoflurane concentration was kept at 2% until the rectal temperature reached 27.5 °C and was reduced to 1%, and then isoflurane inhalation was stopped at 22.5 °C. Using this procedure, the rectal temperature continuously decreased and reached a deep hypothermic condition, i.e., 15 °C (Fig. 1c). The average time to reach 15 °C was 151 ± 7 min (*n* = 6).

To determine whether the deep hypothermia induced using isoflurane is reversible, the hypothermic rats were transferred to a room kept at 22 °C and warmed using a heating lamp. In all rats, shivering and spontaneous locomotion were observed when their rectal temperature became over 20 °C, and then their body temperature recovered (data not shown).

### Changes in heartbeat during induction of deep hypothermia

**Fig. 2:**
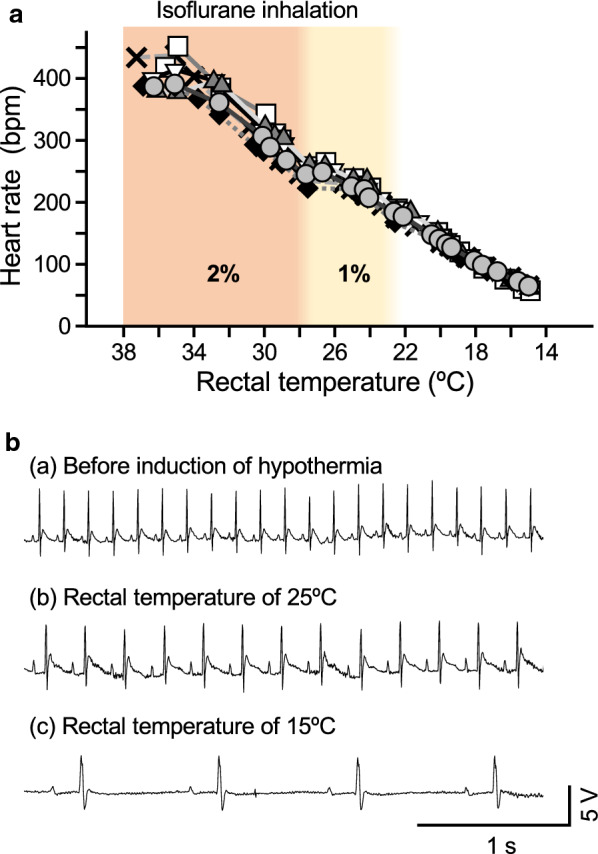
Changes in heartbeat during induction of deep hypothermia. **a** For each rat, heart rate was plotted against rectal temperature (*n* = 6). The heart rate declined concomitantly with the decrease in rectal temperature. **b** Representative ECG tracings that were recorded before induction of hypothermia (**a**) at rectal temperatures of 25 °C (**b**) and 15 °C (**c**). Regular sinus rhythm was fundamentally maintained, and no abnormal sign was recorded on an ECG while the rectal temperature was decreasing

Heart rate declined concomitantly with the decrease in rectal temperature (Fig. 2a). The average heart rate at 15 °C (64 ± 3 beats/min, *n* = 6) was significantly lower than that in a euthermic condition (382 ± 14 beats/min, *n* = 6). Regular sinus rhythm was fundamentally maintained, and no abnormal sign was recorded on an ECG while the rectal temperature was decreasing (Fig. 2b). Before induction of hypothermia, PR interval, QRS duration and QT interval were 45 ± 4, 19 ± 3, 69 ± 9 ms, respectively. When the rectal temperature had reached 15 °C, these parameters were significantly increased to 210 ± 16, 57 ± 21, 221 ± 86 ms, respectively.

### Effects of hexamethonium injection on the induction of deep hypothermia

To investigate the roles of the autonomic nervous system in induction and maintenance of deep hypothermia, we injected hexamethonium (5 mg/kg) intravenously to block sympathetic/parasympathetic ganglionic transmission just after stopping isoflurane inhalation. Injection of the ganglionic blocker had almost no effect on changes in body temperature as well as ECG and heart rate (49 ± 27 beats/min before injection vs 51 ± 30 beats/min after injection, *n* = 4). On the other hand, intravenous injection of hexamethonium (5 mg/kg) significantly decreased heart rate in euthermic rats anesthetized with isoflurane inhalation (326 ± 52 beats/min before injection vs 276 ± 19 beats/min after injection, *p* < 0.05, *n* = 4), suggesting that 5 mg/kg of hexamethonium was an effective dose.

### c-Fos expression in the rat brain during periods of induction and maintenance of deep hypothermia

**Fig. 3:**
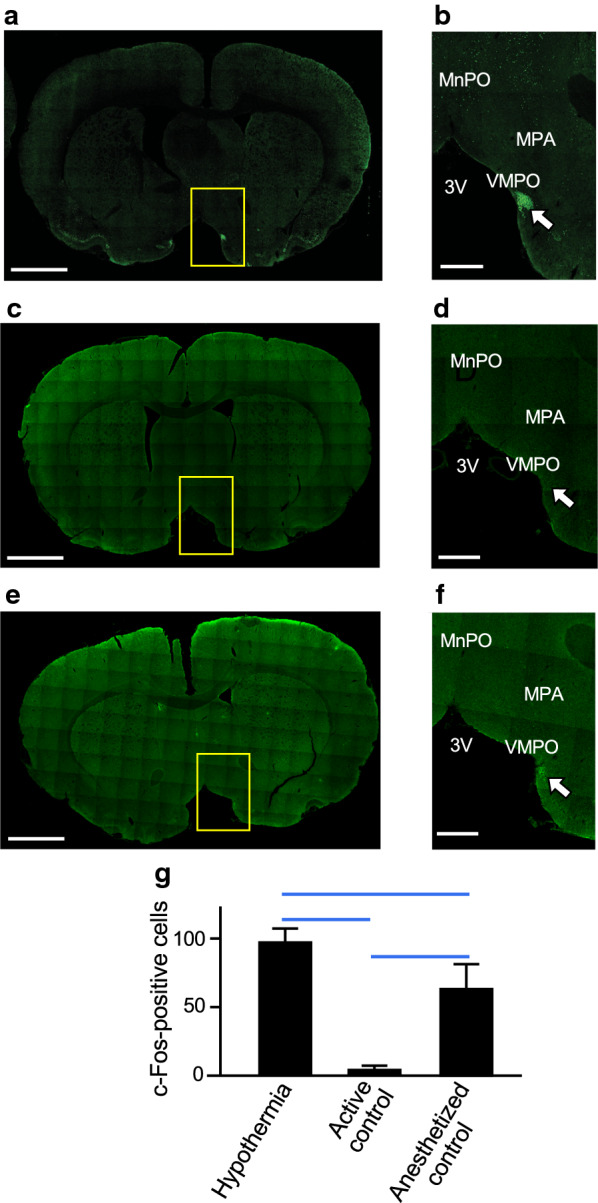
Immunohistochemistry for c-Fos protein in the brain. Representative results of immunohistochemistry for c-Fos protein in the hypothermic rat (**a**, **b**), active control rat (**c**, **d**) and anesthetized control rat (**e**, **f**) are shown. **a**, **c**, **e** a low-magnification view of the brain. Scale bar = 2 mm. **b**, **d**, **f** a high-magnification view of the hypothalamus. Arrows show immunoreactivity for c-Fos protein. In the hypothermic rat, c-Fos-positive cells were confined to the VMPO of the hypothalamus. Scale bar = 500 µm. 3 V, 3rd ventricle; MnPO, median preoptic nucleus, VMPO, ventromedial preoptic nucleus; MPA, medial preoptic area. **g** shows the numbers of c-Fos-positive cells in the VMPO of the hypothermic rat, active control rat and anesthetized control rat. Values are means ± SD (*n* = 3). The blue horizontal lines denote statistical significance (*p* < 0.05)

To identify active brain sites during induction and maintenance of deep hypothermia, we performed immunohistochemistry using antibodies for c-Fos (Fig. 3). As shown in Fig. 3a and b, c-Fos-positive cells were confined to the VMPO of the hypothalamus in the hypothermic rat (rectal temperature of 15 °C). The number of c-Fos-positive cells in the VMPO of the hypothermic rat was significantly larger than that in the active control and anesthetized control rats (Fig. 3g). In addition, the number of c-Fos-positive cells in the VMPO of the anesthetized rat was significantly larger than that in the active rat (Fig. 3g).

### Evaluation of organ injury and dysfunction during periods of induction and/or maintenance of deep hypothermia

**Fig. 4:**
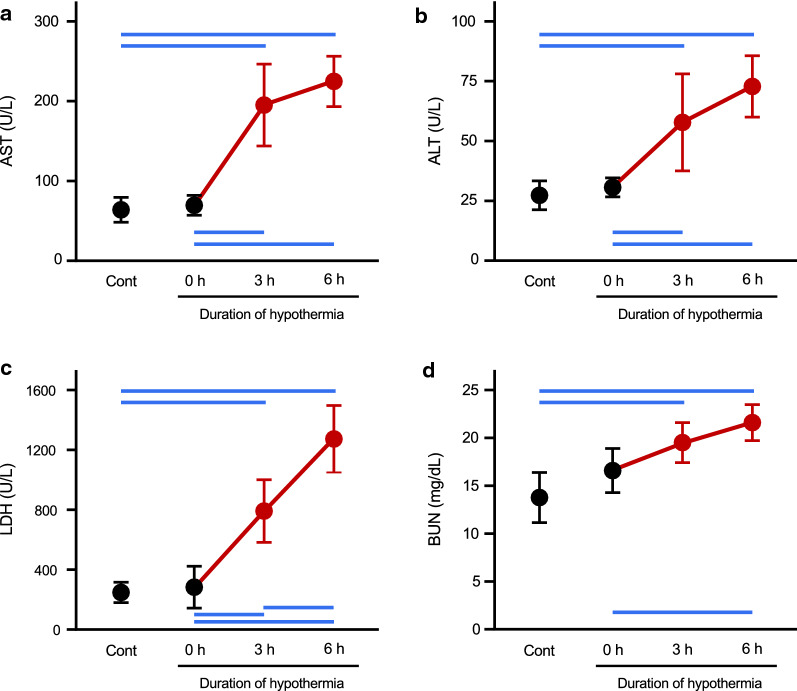
Effects of induction and maintenance of hypothermia on serum levels of organ injury markers. Serum levels of AST, ALT, LDH and BUN are shown in panels **a**–**d**, respectively. *Cont* represents the value in rats before inducing hypothermia. *0* h shows the value in rats just after reaching 15 °C. 3 h and 6 h show the values in rats maintained at 15 °C for 3 h and 6 h, respectively. Comparison between black symbols (*Cont* vs *0 h*) revealed that the process of induction of hypothermia does not affect serum levels of organ injury markers. On the other hand, red symbols (3 h and 6 h) indicate that maintenance of the hypothermic condition significantly increased serum levels of these markers. Values are means ± SD (*n* = 5). The blue horizontal lines denote statistical significance (*p* < 0.05)

We first evaluated how the process of induction of hypothermia affects serum levels of organ injury markers. As shown in Fig. 4a–d, serum levels of AST, ALT, LDH and BUN just after the rectal temperature had reached 15 °C were not significantly different from those in euthermic controls (compare black symbols; control vs 0 h).

Next, deep hypothermia (i.e.*,* rectal temperature of 15 °C) was maintained by placing the hypothermic rats on a cold stainless plate, and the effects of maintenance of the hypothermic conditions were evaluated. The values of all markers were increased significantly after hypothermia had been maintained for 3 h and 6 h (Fig. 4; see red symbols). The values of AST, ALT and BUN at 3 h and those at 6 h were not significantly different, whereas LDH level was significantly higher at 6 h than at 3 h.

### Evaluation of organ injury and dysfunction during recovery from deep hypothermia

**Fig. 5:**
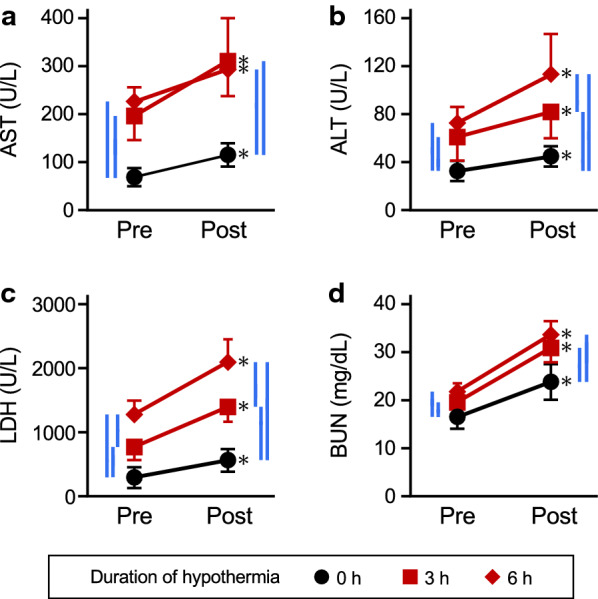
Effects of recovery of body temperature on serum levels of organ injury markers. Serum levels of AST, ALT, LDH and BUN are shown in panels **a**–**d**, respectively. *Pre* and *Post* represent the values in rats before and after recovery of body temperature. Warming the rats immediately after the temperature had reached 15 °C (compare black symbols) showed that the recovery process itself significantly increased serum levels of these organ injury markers. When the rats that had been kept in the hypothermic condition for 3 h or 6 h were warmed and allowed to recover, serum levels of AST, ALT, LDH and BUN, which were increased during the maintenance of hypothermia, were further increased (compare red symbols). Two-way ANOVA analyses showed a significant interaction between the effects of the duration of maintenance of hypothermia and effects of the recovery process. Values are means ± SD (*n* = 5). The blue vertical lines denote statistical significance (*p* < 0.05). Asterisks denote significant difference between *Pre* and *Post* (*p* < 0.05)

Since the process of induction of hypothermia had no obvious effect on organ injury markers (Fig. 5; black symbols), we considered that warming the rats immediately after reaching 15 °C would enable evaluation of the effects of the recovery process. Serum levels of AST, ALT, LDH and BUN after recovery from the deep hypothermia were significantly higher than those before recovery (Fig. 5; see black symbols).

Rats that had been kept in the hypothermic condition for 3 or 6 h were also warmed to examine the interaction between harmful effects of the recovery process and those of the maintenance periods. Serum levels of AST, ALT, LDH and BUN, which were increased during the maintenance of hypothermia, were further increased by undergoing the recovery process (Fig. 5a–d, respectively; see red symbols). Two-way ANOVA analyses showed a significant interaction between the effects of the period of maintenance of hypothermia and the effects of the recovery process (Fig. 5).

### Evaluation of the aftereffects of induction of deep hypothermia

**Fig. 6:**
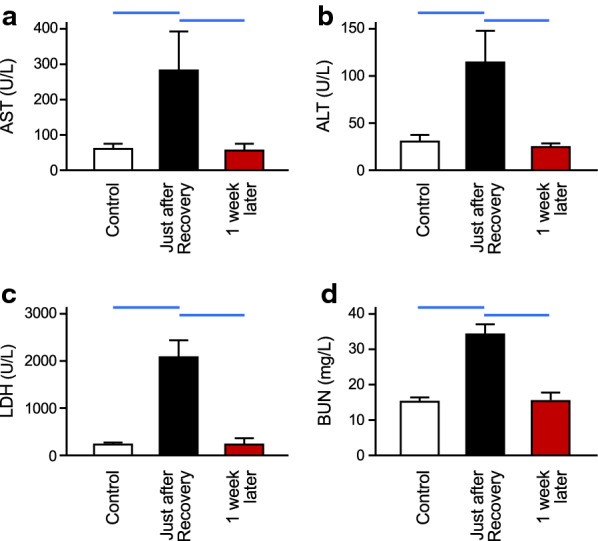
Aftereffects of inducing deep hypothermia in rats. Serum levels of AST, ALT, LDH and BUN are shown in panels **a**–**d**, respectively. *Control* represents the value in rats before inducing hypothermia. *Just after recovery* shows the value in rats that had been kept in the hypothermic condition for 6 h and recovered to a euthermic condition. After recovery, the rats were returned to the animal room and kept there for 1 week. The values in these rats are shown as *1 week later*. The elevated values of organ injury markers just after recovery of rectal temperature were decreased to levels comparable to those before induction of hypothermia (control). Values are means ± SD (*n* = 5). The blue horizontal lines denote statistical significance (*p* < 0.05)

The rats that experienced the hypothermic condition for 6 h were returned to the animal room in which the animals had been kept prior to the experiments. One week later, blood samples were collected to evaluate the aftereffects of induction of deep hypothermia. As shown in Fig. 6a–d, the elevated values of AST, ALT, LDH and BUN just after recovery of rectal temperature were decreased to levels comparable to those before induction of hypothermia (control).

## Discussion

In the present study, we established a novel method for inducing deep hypothermia, resembling hypothermia during hibernation, by isoflurane inhalation and cooling in a non-hibernator, the rat. Our principal findings were (1) cooling rats that had been anesthetized with isoflurane caused a time-dependent decrease in rectal temperature, but cardiac arrest occurred before their body temperature reached 20 °C when isoflurane inhalation was continued throughout the cooling process, (2) deep hypothermia was successfully induced by stopping the inhalation of isoflurane when the rectal temperature of rats reached 22.5 °C, although stopping the inhalation at 27.5 °C resulted in spontaneous recovery of rectal temperature, (3) injection of a ganglionic blocker had almost no effect on body temperature as well as ECG and heart rate during hypothermia, (4) c-Fos-positive cells were markedly detected in the VMPO of the hypothalamus during hypothermia, (5) the hypothermic condition was able to be maintained for up to 6 h, (6) warming the hypothermic rats allowed recovery of rectal temperature, and (7) both the maintenance of and recovery from hypothermia caused organ injury, but the damage was transient and recovered within 1 week. These findings indicate that the established procedure is appropriate for inducing deep hypothermia in a non-hibernator, the rat.

The most remarkable finding of the present study is that removal of anesthetic action by stopping isoflurane inhalation at the appropriate timing is required for inducing deep hypothermia in rats. In natural hibernators, it has been suggested that some specific sites of the brain maintain functional activity during torpor, although deep hypothermia brings about general depression of brain function as evidenced by silencing of cortical electroencephalogram patterns [11]. It seems likely that the active brain sites regulate protective mechanisms to ensure that organs remain undamaged during torpid states [11]. We found that offset of isoflurane inhalation allowed beating of the heart, whereas the presence of anesthetics throughout the cooling process caused cardiac arrest. These findings suggest that the operation of specific brain sites under a deep hypothermic condition is not inherent to hibernators but is reproducible, at least in part, in non-hibernators. The novel method established in the present study may be appropriate for reproducing the beneficial brain state under the condition of deep hypothermia in rats.

We found c-Fos-positive cells in the VMPO, which is a part of the preoptic area (POA), in rats under hypothermia without anesthesia. This is in contrast to the reported observation in thirteen-lined ground squirrels. During torpor of hibernation, a very low signal level of c-Fos immunohistochemistry was detected throughout the entire POA [22]. Interestingly, strong c-Fos expression was detected in the VMPO during the arousal phase of torpor in squirrels [22]. In addition, the VMPO has been reported to be activated by injection of pyrogens such as prostaglandin E2 and lipopolysaccharide in rats [23, 24]. Given these facts, it is natural to assume that a heat-producing reaction is triggered in response to stopping isoflurane inhalation. It should be noted, however, that c-Fos protein usually becomes detectable at 20 to 90 min post-stimulation and its half-life is 90 to 100 min in a euthermic state [25]. Since shivering thermogenesis spontaneously occurred when the isoflurane inhalation was stopped at an early stage of induction of hypothermia (above 27.5 °C), it is thought that the VMPO is activated in this stage. We detected c-Fos-positive cells confined to the VMPO in the hypothermic rat and the number of c-Fos-positive cells in the VMPO of the hypothermic rat was significantly larger than that in the euthermic control rats. It is therefore possible that c-Fos signals detected in the VMPO of deeply hypothermic animals are due to activation of neurons during the early induction phase of hypothermia. In addition, the number of c-Fos-positive cells in the VMPO of the anesthetized rat was significantly larger than that in the active rat. The finding suggests that isoflurane anesthetization itself might also contribute to activation of neurons in the VMPO. Since a deep hypothermic condition was not established in the presence of anesthesia, there was no proper control to determine whether c-Fos expression is promoted after stopping isoflurane inhalation (i.e., deep hypothermia with anesthesia). On the other hand, we cannot answer the question of whether neurons in the VMPO are continuously activated even after a state of deep hypothermia has been reached. Clearly, the timing of VMPO activation is an important issue to be addressed in future investigations.

Our results suggest that the thermoregulatory center may stop working under an extreme hypothermic condition. It is widely accepted that POA, which is located in the rostral pole of the hypothalamus, functions as the thermoregulatory center in many mammals including rats [26]. Cooling of the POA as well as thermosensory neural inputs from skin thermoreceptors to the POA elicit shivering and other heat-generating responses including non-shivering thermogenesis in brown adipose tissue [26]. Inhibition of these thermoregulatory responses is essential for decreasing body temperature artificially. Isoflurane anesthesia is an efficacious strategy for suppressing the thermoregulatory responses as evidenced by the fact that removal of the anesthetic action at a temperature above 27.5 °C elicited shivering thermogenesis. Importantly, shivering was not induced by removing anesthesia after body temperature decreased to less than 22.5 °C. The finding suggests that once the body temperature has entered extremely hypothermic ranges, thermoregulation for cold defense would not operate. Hence, several methods for decreasing body temperature to this range can induce deep hypothermia in non-hibernating animals. For instance, it has been demonstrated that activation of transient receptor potential vanilloid 1, which may accelerate heat loss, brought about deep hypothermia in mice without promoting a shivering response [27].

The rats maintained regular sinus rhythm even after induction of deep hypothermia by the established method (Fig. 2). This is surprising because hypothermia commonly causes cardiac arrhythmias [8] and their frequency and severity are correlated with the depth of hypothermia [7]. We previously reported that induction of artificial hypothermia by pentobarbital anesthesia and cooling in a hibernator, the hamster, caused arrhythmias such as atrioventricular block and ventricular fibrillation [18]. Since abnormal ECGs are not obvious in naturally hibernating animals [18, 28, 29], the occurrence of arrhythmias is not directly caused by an extremely low temperature. Alternatively, it is probable that impairment of the putative protective pathways by anesthesia may be the major cause of the arrhythmias. The mechanism underlying the arrhythmias during hypothermia is thought to be related to a decreased spontaneous depolarization of the sinus node and a slow myocardial impulse conduction [30]. The electrophysiological activities of the sinus node and myocardium are modulated by the autonomic nervous system, and cardiac sympatho-vagal imbalance may cause arrhythmias [31–33]. It is therefore rational to consider that balanced control of cardiac excitability by the autonomic nervous system might be critical for beating of the heart with a regular sinus rhythm during deep hypothermia. However, we found that the application of an autonomic ganglion blocker had little effect on ECG during deep hypothermia. The autonomic nervous system might therefore play minor roles in the regulation of sinus rhythm during hypothermia, whereas removal of anesthetic effects is essential for maintaining regular sinus rhythm under the condition of deep hypothermia in rats.

The deep hypothermia induced by isoflurane anesthesia and cooling does not result in severe organ injury. We measured serum levels of AST, ALT and LDH as indicators of the onset of organ injury [34–36] and BUN as an indicator of a disorder of renal function [37]. Interestingly, all of these parameters showed no change when body temperature reached 15 °C. These results suggest that the reduction process does not have a harmful impact. In contrast, both maintenance of deep hypothermia and recovery from hypothermia cause organ injury to some extent. Two-way ANOVA showed a significant interaction between effects of maintenance of the duration and effects of recovery. This indicates that prolonged duration of deep hypothermia increases the risk during the recovery process. However, in rats that recovered after the maintenance of hypothermia for 6 h, we found that the elevated values of serum parameters returned to normal levels within 1 week. These results indicate that adverse effects of maintenance of and recovery from deep hypothermia would be transient and not cause severe sequela. Notably, the method for inducing hypothermia in this study is not accompanied by the central injection of drugs. This would be highly beneficial for using deep hypothermia in the clinical field.

## Conclusions

In summary, the present study showed that it is possible to induce deep hypothermia in non-hibernators, at least in rats, by removing the inhalation anesthetics at the appropriate timing. The novel method established in this study may be valuable for the clinical application of hypothermia since the procedure is simple and does not require central manipulation.

## Data Availability

The datasets used and analyzed during the current study are available from the corresponding author on reasonable request.

## Abbreviations

A1AR: Adenosine A1 receptors
ALT: Alanine aminotransferase
ANOVA: Analysis of variance
AST: Serum aspartate aminotransferase
BUN: Blood urea nitrogen
ECG: Electrocardiogram
icv: Intracerebroventricular
LDH: Lactate dehydrogenase
MnPO: Median preoptic nucleus
MPA: Medial preoptic area
POA: Preoptic area
SD: Standard deviation
VMPO: Ventromedial preoptic nucleus
3 V: 3Rd ventricle
